# 

**Published:** 1895-04-27

**Authors:** 


CONTENTS.

The Nation and Its Paralytics and Epileptics - - 55
The Education of Nervous Children - - - - - 56
Pulmonary Congestion and Its Treatment.—I. By Sir B. W. Richardson, M.D., F.R.S. - - - - - - - - 57
Medical Progress and Hospital Clinics—
Note on Blood-letting Without Loss of Blood - - - - 61
Treatment of Hypertrophy of the Prostate at the Northampton General Infirmary - - - - - - - - 62
Progress in Medicine.—Dietetics - - - - - - 62
Progress in Surgery.—Surgery of the Intestines - - - 64
New Appliances and Things Medical - - - - - 65
A Byzantine Clinical History - - - - - - - - 58
Annotations.—The Doctor on Guard—An Ambulance System for Paris—"Biliousness" - - - - - - - - - - 59
Notes and News - - - - - - - - - - - - 60
The Institutional Workshop:
Hospital Construction—The Poplar Hospital for Accidents - 67
The Story of the Insane from Year to Year - - - - 68
Practical Departments - - - - - - - - - 69
Construction Notes - - - - - - - - - - 70
Scraps and Gleanings - - - - - - - - - 70
The Book World of Medicine and Science - - - - 71

EXTRA SUPPLEMENT.—THE NURSING MIRROR.

News from the Nursing World.—A Favoured Hospital—The National Hospital—Hampstead Hospital—A Distinguished Nurse—Nurses in Carlisle—In Palestine—Women Lecturers—Royal British Nurses' Association—Night Nursing Sanctioned —The Matrons' Council—Nursing in Bedfordshire—Wasted Lives—Two Nurses at Johannesburg—Short Items - - - - - - - - - - - - - xxiii
Elementary Anatomy and Surgery for Nurses - - xxv
Appointments - - - - - - - - - - - xxv
Medical Stores for Madagascar - - - - - - - xxv
Nursing in America—The Convention of Superintendents and Members of the Association - - xxvi
Everybody's Opinion - - - - - - - - - xxvii
Royal British Nurses' Association - - - - - - xxvii
Where to Go - - - - - - - - - - - xxvii
Novelties for Nurses—Minor Appointments - - - xxvii

